# Trends in Apgar scores and umbilical artery pH: a population-based cohort study on 10,696,831 live births in Germany, 2008–2022

**DOI:** 10.1007/s00431-024-05475-w

**Published:** 2024-02-17

**Authors:** Vinzenz Boos, Christoph Bührer

**Affiliations:** 1 Department of Neonatology, Newborn Research, University Hospital Zurich (USZ), University of Zurich (UZH), Frauenklinikstrasse 10, 8091 Zurich, Switzerland; 2 Department of Neonatology, Charité - Universitätsmedizin Berlin, Charitéplatz 1, 10117 Berlin, Germany

**Keywords:** Apgar scores, *E*-value, Base excess, Time trends, Umbilical arterial blood pH

## Abstract

Low Apgar scores and low umbilical arterial (UA) blood pH are considered indicators of adverse perinatal events. This study investigated trends of these perinatal health indicators in Germany. Perinatal data on 10,696,831 in-hospital live births from 2008 to 2022 were obtained from quality assurance institutes. Joinpoint regression analysis was used to quantify trends of low Apgar score and UA pH. Additional analyses stratified by mode of delivery were performed on term singletons with cephalic presentation. Robustness against unmeasured confounding was analyzed using the *E*-value sensitivity analysis. The overall rates of 5-min Apgar scores < 7 and UA pH < 7.10 in liveborn infants were 1.17% and 1.98%, respectively. For low Apgar scores, joinpoint analysis revealed an increase from 2008 to 2011 (annual percent change (APC) 5.19; 95% CI 3.66–9.00) followed by a slower increase from 2011 to 2019 (APC 2.56; 95% CI 2.00–3.03) and a stabilization from 2019 onwards (APC − 0.64; 95% CI − 3.60 to 0.62). The rate of UA blood pH < 7.10 increased significantly between 2011 and 2017 (APC 5.90; 95% CI 5.15–7.42). For term singletons in cephalic presentation, the risk amplification of low Apgar scores was highest after instrumental delivery (risk ratio 1.623, 95% CI 1.509–1.745), whereas those born spontaneous had the highest increase in pH < 7.10 (risk ratio 1.648, 95% CI 1.615–1.682).

*          Conclusion*: Rates of low 5-min Apgar scores and UA pH in liveborn infants increased from 2008 to 2022 in Germany.

**Table:** 

**What is Known:** *• Low Apgar scores at 5 min after birth and umbilical arterial blood pH are associated with adverse perinatal outcomes.* *• Prospective collection of Apgar scores and arterial blood pH data allows for nationwide quality assurance.*	
**What is New:** *• The rates of liveborn infants with 5-min Apgar scores < 7 rose from 0.97 to 1.30% and that of umbilical arterial blood pH < 7.10 from 1.55 to 2.30% between 2008–2010 and 2020–2022. * *• In spontaneously born term singletons in cephalic presentation, the rate of metabolic acidosis with pH < 7.10 and BE < −5 mmol/L in umbilical arterial blood roughly doubled between the periods 2008–2010 and 2020–2022.*

**What is Known:**

*• Low Apgar scores at 5 min after birth and umbilical arterial blood pH are associated with adverse perinatal outcomes.*

*• Prospective collection of Apgar scores and arterial blood pH data allows for nationwide quality assurance.*

**What is New:**

*• The rates of liveborn infants with 5-min Apgar scores < 7 rose from 0.97 to 1.30% and that of umbilical arterial blood pH < 7.10 from 1.55 to 2.30% between 2008–2010 and 2020–2022. *

*• In spontaneously born term singletons in cephalic presentation, the rate of metabolic acidosis with pH < 7.10 and BE < −5 mmol/L in umbilical arterial blood roughly doubled between the periods 2008–2010 and 2020–2022.*

## Introduction

The Apgar score has been used for 70 years for assessing the vitality of neonates and the effectiveness of resuscitative efforts immediately after birth. Elements of the Apgar score include skin color, heart rate, reflexes, muscle tone, and respiration [1]. The Apgar score at 5 min has become a widely used standardized tool for neonatal assessment and an indicator for neonates at risk for developing hypoxia–ischemia-related neonatal encephalopathy [2–4]. Low Apgar scores have been associated with neonatal death and long-term neurological impairment such as cerebral palsy, epilepsy, sensorineural defects, intellectual disability, and attention deficit hyperactivity disorder [5–11]. The 5-min Apgar score emerged as a widely accepted perinatal health indicator in national and international statistics [12, 13].

However, Apgar scoring is heavily prone to bias. Originally designed to be performed by an independent observer, the assessment may in practice be done in retrospect by staff who took care of the infant [1]. There is poor interobserver agreement, and Apgar scoring makes no allowance for various intensities of respiratory support or the decreased reflex and muscle tone that come along with prematurity [4, 14–17]. In an effort to complement Apgar scoring with an objective indicator of fetal compromise, umbilical arterial (UA) cord blood pH has been introduced as a marker of acute perinatal events that can lead to neurological injury [18–20]. A low UA pH has been associated with neonatal mortality, neonatal encephalopathy, and cerebral palsy [21]. Metabolic acidosis in particular appeared to be associated with adverse outcomes, with base excess (BE) being a significant marker for fetal metabolic acidemia [22, 23].

The Apgar score and UA pH have both been routinely used to diagnose acute peripartum or intrapartum hypoxic-ischemic events which may lead to neonatal encephalopathy [3, 4]. Most trials on therapeutic hypothermia, a treatment that increases survival without increasing major disability in term and late preterm infants with moderate or severe encephalopathy and evidence of intrapartum asphyxia, have applied Apgar scores and UA pH as criteria for peripartum asphyxia [24]. While the Apgar score is routinely used after birth, the determination of UA blood pH is not yet so widespread, or is only recommended in certain situations [4, 25]. However, fetal acidosis with a higher risk of adverse outcomes was also present in neonates with a 5-min Apgar score ≥ 7, demonstrating that clinical aspects alone are not sufficient to assess the neonate [4].

Although perinatal health data including Apgar scores are regularly published, there has been no analysis of trends in Apgar scores or UA pH values in Germany. The aim of this study was to investigate temporal trends in Apgar scores, UA pH, and BE as indicators of birth asphyxia over the past 15 years (2008–2022) in Germany.

## Methods

### Population and data

For the purpose of this population-based cohort study, aggregate data of federal quality assurance reports (“German Perinatal Reports “) covering the years 2008 to 2022 were used. Collection of perinatal data has been mandatory for all in-hospital deliveries in Germany in the context of statutory quality assurance. The perinatal reports were published annually by the institutes entrusted with the national data collection (2009–2014: aQua—Institut für angewandte Qualitätsförderung und Forschung im Gesundheitswesen, Göttingen, Germany; 2015–2022: Institut für Qualität und Transparenz im Gesundheitswesen, Berlin, Germany). They are annually revised and contain data on mothers and their infants. Most data were extracted from these reports. The total number of live births in Germany, including home births, was extracted from the “Genesis-Online” database of the Federal Statistical Office of Germany [26].

As completely de-identified, publicly available aggregate data were used for this study; no ethical approval was required.

### Outcome measurements

Outcomes of interest were the Apgar score at 5 min after birth and UA blood values among in-hospital liveborn infants. Primary outcomes were the incidence of Apgar score < 7 and the UA pH < 7.10, respectively. The Apgar score was additionally analyzed for subgroups with scores of 0, 1–3, 4–6, 7–9, and 10; the UA cord blood pH for subgroups with thresholds of 7.00, 7.10, and 7.20; and the UA BE for subgroups with thresholds of − 10 mmol/L, − 5 mmol/L, and 0 mmol/L, respectively. Temporal trends for Apgar < 7 and UA pH < 7.10 were calculated for the entire study period. In addition, risk ratios for the first (2008–2010) compared to the last (2020–2022) 3 years of the study period were computed for subgroups of Apgar scores, UA pH, and BE.

### Term singleton neonates with cephalic presentation

Data on all liveborn term singletons with cephalic presentation (TSC) delivered in-hospital were further analyzed to account for preterm and postterm birth, multiple pregnancies, and fetal presentation as potential confounders on the observed trends of the entire study cohort. Incidence and risk ratios for Apgar scores, UA pH, and BE between the 2008–2010 and 2020–2022 periods were calculated accordingly, stratified by mode of delivery.

### Statistics

The Mann–Kendall trend test was used to detect trends regarding data completeness. A joinpoint regression model was used to explore potential temporal patterns of the main outcomes. Joinpoint analysis identified infliction points where linear trends changed significantly. The number of joinpoints was selected based on permutation tests with significance level *α* = 0.05 and the weighted Bayesian information criterion. For each trend line, the annual percent change (APC) with associated confidence intervals (CI) was calculated. Risk ratios (RR) with 95% CI were computed to compare outcome rates between the periods 2008–2010 and 2020–2022. In sensitivity analysis, *E*-values and their confidence interval limits closer to the null were calculated to assess the robustness of the association between time and outcomes to potential unmeasured or unadjusted confounding [27]. All statistical analyses were conducted using Joinpoint Regression Program, version 5.0.2 (Statistical Methodology and Applications Branch, in Surveillance Research Program, National Cancer Institute, USA) and RStudio v2023.09.1 + 494 (RStudio, Boston, MA) in R, version 4.3.1 (The R Foundation for Statistical Computing, Vienna, Austria).

## Results

### Study population

The quality assurance reports contained data on 10,696,831 in-hospital live births during the period 2008 to 2022 in Germany, which accounted for 97.72% of all 10,946,497 live births registered in the database of the Federal Statistical Office of Germany. There was no time trend regarding in-hospital deliveries (median 97.85%, IQR 97.33–98.12%; *p* = 0.488) reported by the quality assurance institutes.

Apgar scores at 5 min were reported for 10,653,757 (99.60%) of these infants, and UA blood pH analyses were available for 10,576,047 (98.87%), respectively. The total number of UA BE values available for analysis could not be assessed, as positive BE values were not reported. Among all live births, there were 8,644,157 (80.81%) TSC neonates.

### Trends in outcome measurements

**Fig. 1:**
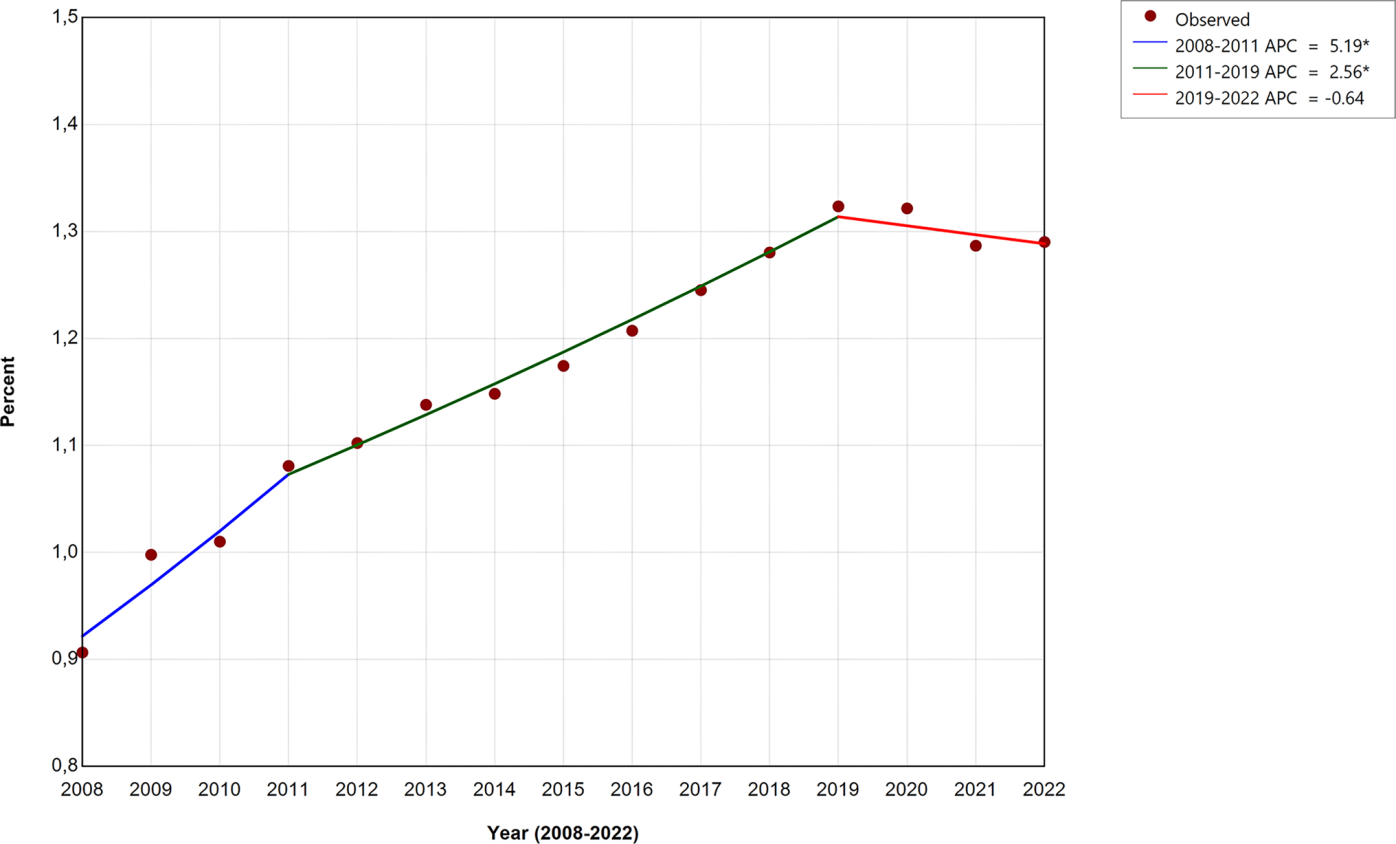
Incidence of 5-min Apgar scores < 7. Temporal patterns of the annual rate of neonates with a 5-min Apgar score < 7 per 100 in-hospital live births (continuous line). Apparent breakpoints marking a change of the slope emerged for 2011 and 2019. Asterisk (*) indicates that the annual percent change (APC) is significantly different from zero at the alpha = 0.05 level

An Apgar score < 7 at 5 min of life was present in 125,648 (1.17%) of all patients and increased from 0.97% in 2008–2010 to 1.30% in 2020–2022 (Table 1). The evolution of the incidence of this outcome measure was not monotonic (Fig. 1). The best-fitting model using joinpoint regression included two break points in 2011 and 2019 and revealed significant increases between 2008 and 2019. The rate of low Apgar scores significantly increased over time (APC 5.19; 95% CI 3.66–9.00) between 2008 and 2011 and at a slower pace (APC 2.56; 95% CI 2.00–3.03) between 2011 and 2019. From 2019 onwards, the incidence remained constant (APC − 0.64; 95% CI − 3.60 to 0.62).

**Table 1:** Trends in Apgar scores and umbilical arterial blood pH in in-hospital liveborn infants in Germany

	**2008–2010**		**2020–2022**			
	**(*n* = 1,975,607)**		**(*n* = 2,242,290)**			
	***n***	**Rate (%)**	***n***	**Rate (%)**	**Risk ratio (95% CI)**	***E*-value (95% CL)**
**Primary outcomes**						
5-min Apgar score < 7	19,183	0.97	29,140	1.30	1.338 (1.314–1.363)	2.011 (1.957)
Umbilical arterial blood pH < 7.10	30,612	1.55	51,613	2.30	1.486 (1.465–1.506)	2.335 (2.290)
**Subgroups**						
*Apgar scores at 5 min*						
Apgar score 0	1052	0.05	994	0.04	0.832 (0.763–0.908)	1.693 (1.436)
Apgar score 1–3	3104	0.16	4482	0.20	1.272 (1.215–1.332)	1.861 (1.727)
Apgar score 4–6	15,027	0.76	23,664	1.06	1.387 (1.360–1.416)	2.121 (2.059)
Apgar score 7–9	430,485	21.79	470,811	21.00	0.964 (0.960–0.967)	1.236 (1.221)
Apgar score 10	1,516,955	76.78	1,735,393	77.39	1.008 (1.007–1.009)	1.097 (1.090)
Apgar score N/A	8984	0.45	6946	0.31	0.681 (0.660–0.703)	2.297 (2.198)
*Umbilical arterial blood*						
pH < 7.00	3939	0.20	5867	0.26	1.312 (1.260–1.366)	1.953 (1.833)
pH 7.00–7.09	26,673	1.35	45,746	2.04	1.511 (1.489–1.534)	2.390 (2.341)
pH 7.10–7.19	201,165	10.18	333,744	14.88	1.462 (1.454–1.469)	2.283 (2.267)
pH ≥ 7.20	1,702,923	86.20	1,837,651	81.95	0.951 (0.950–0.952)	1.285 (1.282)
pH N/A	40,907	2.07	19,282	0.86	0.415 (0.408–0.422)	4.249 (4.166)
BE < − 10 mmol/L	69,374	3.51	115,685	5.16	1.469 (1.456–1.483)	2.300 (2.270)
BE − 10 to < − 5 mmol/L	417,015	21.11	676,427	30.17	1.429 (1.424–1.434)	2.212 (2.202)
BE − 5 to 0 mmol/L	1,019,843	51.62	1,189,977	53.07	1.028 (1.026–1.030)	1.198 (1.190)
BE > 0 or N/A	469,375	23.76	260,201	11.60	0.488 (0.486–0.491)	3.512 (3.493)

*BE* base excess, *CI* confidence interval, *CL* confidence limit, *N/A* not available

**Fig. 2:**
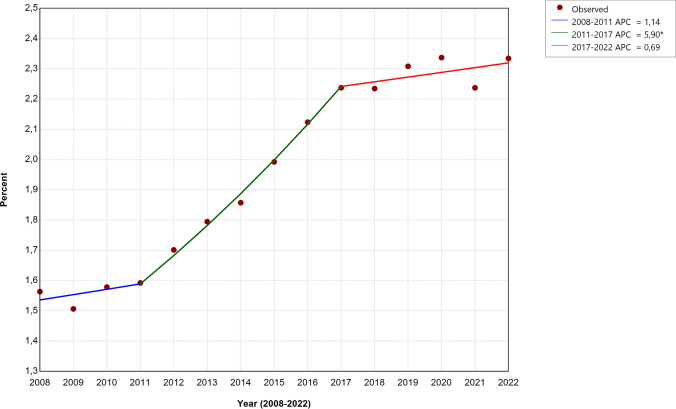
Incidence of umbilical arterial blood pH < 7.10. Temporal patterns of the annual rate of neonates with umbilical arterial blood pH < 7.10 per 100 in-hospital live births (continuous line) with breakpoints (2011, 2017) where the slope changed. Asterisk (*) indicates that the annual percent change (APC) is significantly different from zero at the alpha = 0.05 level

An UA blood pH < 7.10 was detected in 211,677 (1.98%) cases and increased from 1.55 to 2.30% between 2008–2010 and 2020–2022 (Table 1). Two infliction points were identified for the time trend of UA blood pH < 7.10 (Fig. 2). After a stable period from 2008 to 2011 (APC 1.14; 95% CI − 1.89 to 3.23), the rate of low UA blood pH increased (APC 5.90; 95% CI 5.15–7.42) until 2017 and flattened on a higher plateau between 2017 and 2022 (APC 0.69; 95 CI − 0.25 to 1.54).

The average APC for the entire study period was 2.42 (95% CI 2.08–2.84) and 2.99 (95% CI 2.72–3.31) for an Apgar score < 7 and UA blood pH < 7.10, respectively.

Risk for Apgar scores of 0 decreased, while the largest increase between 2008–2010 and 2020–2022 was detected in the group of neonates with Apgar scores 4–6 (RR 1.387, 95% CI 1.360–1.416). The rate of infants with pH < 7.00, 7.00–7.09, and 7.10–7.19 increased, with the greatest increase in neonates with UA pH 7.00–7.09 (RR 1.511, 95% CI 1.489–1.534). In contrast, the rate of neonates with pH ≥ 7.20, by far the largest subgroup, decreased (RR 0.951, 95% CI 0.950–0.952). The analysis of UA BE values revealed the highest risk increase in the subgroup of infants with BE <  − 10 mmol/L (RR 1.469, 95% CI 1.456–1.483).

### Trends in TSC cohort

**Fig. 3:**
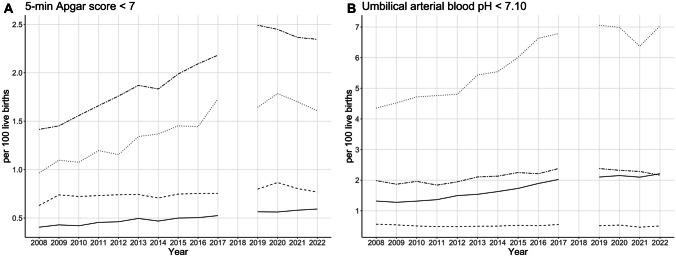
Apgar score and umbilical arterial blood pH in term singletons with cephalic presentation. Time trends for **A** 5-min Apgar score < 7 and **B** umbilical arterial blood pH < 7.10 rates in term singletons with cephalic presentation, stratified by mode of delivery: spontaneous delivery (solid), primary caesarean section (dashed), secondary caesarean section (two dash), and instrumental delivery (dotted). Data for the year 2018 were not available for analysis

Analysis of Apgar score < 7, UA pH, and BE trends in TSC revealed heterogeneous patterns (Table 2, Fig. 3). After stratification by mode of delivery, an increase in the rate of Apgar scores < 7 was observed in all TSC cohorts. Infants born by secondary caesarean section (CS) and instrumental delivery had the largest increases in the risk of a low Apgar score. In the numerically largest group of spontaneous deliveries, the rate of neonates with pH < 7.10 increased significantly. The risk of a pH < 7.10 in combination with both a base excess under − 10 mmol/L (the most severe form of metabolic acidosis in this analysis) and between − 5 and − 10 mmol/L roughly doubled between the periods 2008–2010 and 2020–2022. In addition, there was a significant increase in the risk of being born with a pH < 7.10 after both secondary CS and instrumental delivery. An increase in neonates with pH < 7.10 and a BE <  − 10 mmol/L was particularly evident in infants born by instrumental delivery.

**Table 2:** Trends in Apgar scores and umbilical arterial pH and base excess in term singletons in cephalic presentation, stratified by mode of delivery

	**2008–2010**		**2020–2022**			
	**(*n* = 1,580,243)**		**(*n* = 1,831,910)**			
	***n***	**Rate (%)**	***n***	**Rate (%)**	**Risk ratio (95% CI)**	***E*-value (95% CL)**
**Spontaneous delivery**	(*n* = 1,085,018)		(*n* = 1,228,178)			
5-min Apgar score < 7	4573	0.42	7104	0.58	1.383 (1.333–1.436)	2.111 (1.999)
pH < 7.10	14,166	1.31	26,433	2.15	1.648 (1.615–1.682)	2.682 (2.612)
BE < − 10 mmol/L	7981	0.74	17,436	1.42	1.930 (1.880–1.982)	3.270 (3.166)
BE − 10 to < − 5 mmol/L	3623	0.33	8212	0.67	2.002 (1.926–2.082)	3.419 (3.261)
BE − 5 to 0 mmol/L	511	0.05	553	0.05	0.956 (0.848–1.078)	1.265 (1.000)
pH 7.10–7.19	129,349	11.92	226,152	18.41	1.545 (1.535–1.554)	2.462 (2.441)
**Caesarean section, primary**	(*n* = 170,563)		(*n* = 203,959)			
5-min Apgar score < 7	1189	0.70	1658	0.81	1.166 (1.083–1.256)	1.606 (1.382)
pH < 7.10	916	0.54	1025	0.50	0.936 (0.856–1.023)	1.339 (1.000)
BE < − 10 mmol/L	456	0.27	441	0.22	0.809 (0.710–0.922)	1.777 (1.388)
BE − 10 to < − 5 mmol/L	249	0.15	486	0.24	1.632 (1.401–1.901)	2.648 (2.151)
BE − 5 to 0 mmol/L	51	0.03	76	0.04	1.246 (0.874–1.777)	1.800 (1.000)
pH 7.10–7.19	4049	2.37	4781	2.34	0.987 (0.947–1.029)	1.126 (1.000)
**Caesarean section, secondary**	(*n* = 175,563)		(*n* = 211,222)			
5-min Apgar score < 7	2593	1.48	5041	2.39	1.616 (1.542–1.694)	2.613 (2.456)
pH < 7.10	3405	1.94	4768	2.26	1.164 (1.114–1.216)	1.601 (1.471)
BE < − 10 mmol/L	2055	1.17	3061	1.45	1.238 (1.171–1.309)	1.781 (1.619)
BE − 10 to < − 5 mmol/L	703	0.40	1464	0.69	1.731 (1.582–1.893)	2.856 (2.542)
BE − 5 to 0 mmol/L	106	0.06	195	0.09	1.529 (1.207–1.937)	2.428 (1.707)
pH 7.10–7.19	11,284	6.43	14,941	7.07	1.101 (1.075–1.127)	1.433 (1.359)
**Instrumental delivery**	(*n* = 99,235)		(*n* = 136,073)			
5-min Apgar score < 7	1039	1.05	2312	1.70	1.623 (1.509–1.745)	2.628 (2.385)
pH < 7.10	4500	4.53	9241	6.79	1.498 (1.447–1.550)	2.361 (2.250)
BE < − 10 mmol/L	2701	2.72	6482	4.76	1.750 (1.675–1.829)	2.896 (2.737)
BE − 10 to < − 5 mmol/L	1137	1.15	2517	1.85	1.614 (1.506–1.731)	2.610 (2.379)
BE -5 to 0 mmol/L	111	0.11	158	0.12	1.038 (0.814–1.323)	1.237 (1.000)
pH 7.10–7.19	23,570	23.75	44,946	33.03	1.391 (1.372–1.410)	2.128 (2.087)

*BE* base excess, *CI* confidence interval, *CL* confidence limit

### Sensitivity analysis

The corresponding *E*-value for a 5-min Apgar score < 7 was 2.011 (95% CL 1.957) and for pH < 7.10 in UA cord blood 2.335 (95% CL 2.290), respectively (Table 1). High *E*-values for RR for metabolic acidosis with pH < 7.10 and BE <  − 10 mmol/L (3.270, 95% CL 3.166) and BE − 10 to <  − 5 (3.419, 95% CL 3.261) in the largest cohort of spontaneously delivered TSC neonates indicated that relatively strong unmeasured confounding would be required to explain the entirety of the effect (Table 2).

## Discussion

Using the most up-to-date national quality assurance datasets, this study found that adverse perinatal outcomes as measured by low 5-min Apgar scores and UA pH have increased significantly during the 2010s decade. The observation that the risk of metabolic acidosis in UA blood in spontaneously born term singletons approximately doubled during the study period seemed particularly worrying.

Data on recent trends in Apgar scores are scarce, even more so for UA pH. A recently published study reported that the rate of term singletons with a 5-min Apgar score of < 7 increased by 36.5% between 2010 and 2019 in the Netherlands [28]. The present study made the same observation in a very similar time period, with the risk of an Apgar score < 7 increasing by 33.8% in Germany in the period 2020–2022 compared to 2008–2010. While the population studied by Tacke et al. included only singletons born between 35 and 42 completed gestational weeks, all live births were included in the analysis in Germany. However, the sub-analysis of the TSC neonates allowed a certain comparability of the populations and results. Similar trends also emerged between the Netherlands and Germany regarding fetal acidosis, but these results are only comparable to a limited extent, as UA pH values were only available in 9.6% of live births in the cohort studied by Tacke et al., compared to 98.87% in the German cohort [28]. A study from Denmark reported no change in Apgar scores < 7, but a non-significant decrease in UA cord pH < 7.00 from 2009 to 2015 during implementation of a comprehensive mandatory cardiotocography education program for Danish midwives and physicians [29].

Further studies described increases in adverse neonatal outcomes in the more distant past. Between 2004 and 2010, an increase of 2.8% of neonates with 5-min Apgar < 7 has been reported among European countries [30]. In Norway, the proportion of liveborn infants with 5-min Apgar scores less than 4 and 7 increased between 1999–2000 and 2007–2008, followed by a decrease (< 4) or stabilization (< 7) of these rates between 2007–2008 and 2017–2018, respectively [31].

The separate analysis of TSC neonates enabled analysis of more homogeneous cohorts comprising a large part of the study population and excluding factors such as prematurity and multiple pregnancies. Increases in the rates of infants with low Apgar scores or UA pH values are particularly relevant in term infants, as neither perinatal health indicator has been associated with neurodevelopmental outcomes in extremely preterm or very low birth weight infants, respectively [32, 33]. Within the TSC cohort, a relevant influence of delivery modes on changes in neonatal outcome rates could be demonstrated. While the relative risk of an Apgar score < 7 increased the most in infants born by instrumental delivery between 2008–2010 and 2020–2022, the increase in absolute numbers was highest after spontaneous births and secondary CS. The increase of neonates with UA pH < 7.10, especially those with the lowest category of base excess (< − 10 mmol/L), was highest in relative and absolute terms in spontaneously born infants. In the cohort from the Netherlands, infants born by instrumental vaginal delivery and emergency CS had the highest relative increase in the risk for a 5-min Apgar < 7, comparable to the present study [28]. In Norway, the incidence of low Apgar scores was highest in the period with the highest CS rate; however, no separate analysis by mode of delivery was reported in this cohort [31]. The results in TSC indicated that the risk for developing long-term adverse outcomes such as neurodevelopmental disorders increased for a relevant number of spontaneously delivered full-term infants in Germany.

The use of Apgar scores to determine trends in population-based cohorts is not unproblematic. The Apgar score is a subjective parameter and has a high interobserver variability [14, 15]. There is no accepted standard for reporting Apgar scores in neonates undergoing resuscitation, and neonatal interventions such as mechanical ventilation may affect assignment of the score [4, 16]. The Apgar score cannot be readily applied to preterm infants, as Apgar scores have been shown to be inversely related to gestational age and birth weight [17]. This study attempted to counter this by separately analyzing trends in Apgar scores for term singletons. Despite its disadvantages, the 5-min Apgar score is recommended for neonatal assessment and a widely used perinatal health indicator [3, 4, 12, 13].

The selection of a second indicator for adverse perinatal outcomes, UA pH, was intended to compensate for the aforementioned disadvantages of the Apgar score. A low UA pH was also associated with neurological morbidity and mortality and is commonly used to diagnose perinatal asphyxia [3, 4, 21]. As laboratory parameters of a blood gas analysis, the UA pH value and the BE are objectively measured parameters. However, their use requires correct pre-analytical sample handling, although reports on the stability of UA blood gas analyses provided contradictory results on the clinical relevance of correct pre-analysis for UA values [34–36]. As timing of gas sampling during delayed cord clamping can affect UA blood acid–base parameters, a potential rise in delayed cord clamping practices during the study period may have contributed to minor changes in pH and BE values [37].

The Apgar score and UA pH are both rapidly available indicators for birth asphyxia. Although there may be some degree of correlation between the two parameters, their extent and clinical significance are still unclear [38–41]. A combination of Apgar score and UA pH could have the potential to better identify neonates at risk, as infants with reassuring Apgar scores have been shown to have a residual risk of acidemia [40, 41].

The underlying causes for the increase of low pH and UA pH during the 2010s in Germany remained unclear. Maternal morbidities such as haemorrhagic and hypertensive disorders, advanced maternal age, and obesity have been reported to be associated with low Apgar scores at 5 min or fetal acidosis [42–45]. Whether and to what extent such factors influenced the increase in adverse neonatal outcomes in Germany remained unclear. In the cohort studied, rates for low Apgar score and UA pH remained constant during the COVID-19 pandemic years. Although the pandemic had a profound impact on maternal and fetal health in recent years, there is no evidence that the pandemic has affected Apgar scores or umbilical cord blood pH values compared to pre-pandemic cohorts [46]. Missing data may have impacted the results, particularly if UA blood gas analyses were missing more frequently in compromised neonates when sampling was not prioritized in resuscitation scenarios [47]. However, the almost contemporaneous increase in the risk of neonates being born with a low Apgar score or a low UA pH indicated that there was an actual increase in infants born in poor condition during the 2010s in Germany. Sensitivity analysis suggested that unmeasured confounders were unlikely to explain the entirety of the effect.

This study has several limitations. Although the data from the quality assurance institutes was very comprehensive, it was not 100% complete, which may have had an impact on rare events. The data quality was dependent on the quality of data collection by healthcare professionals, which was not necessarily flawless. The lack of detailed data on births in 2018 made an overall analysis of the TSC cohort during the 15-year study period impossible. The analysis of aggregated data did not allow an analysis of the influence of maternal risk factors on the increase in adverse perinatal outcomes, nor whether the higher rates of neonates with low Apgar or pH led to higher mortality rates.

In conclusion, the rate of adverse perinatal outcomes, as measured by the 5-min Apgar score and UA blood pH, has substantially increased in Germany during the 2010s decade and remained constant on a higher level since then. An almost identical trend was reported for the Netherlands in roughly the same period [28]. The increase of neonates with signs of perinatal depression observed in Germany puts several thousand infants per year at risk of unfavorable neurodevelopmental outcomes. The potential clinical and economic burden of this development warrants further studies to investigate whether this phenomenon also occurred in other high-income countries.

## Data Availability

The manuscript is based on published aggregate data which can be provided upon request. There are no individual patient data to be shared.

## Abbreviations

APC: Annual percent change
BE: Base excess
CS: Caesarean section
RR: Risk ratios
TSC: Term singletons with cephalic presentation
UA: Umbilical arterial
